# European Flint Landraces Grown *In Situ* Reveal Adaptive Introgression from Modern Maize

**DOI:** 10.1371/journal.pone.0121381

**Published:** 2015-04-08

**Authors:** Elena Bitocchi, Elisa Bellucci, Domenico Rau, Emidio Albertini, Monica Rodriguez, Fabio Veronesi, Giovanna Attene, Laura Nanni

**Affiliations:** 1 Department of Agricultural, Food and Environmental Sciences, Università Politecnica delle Marche, Ancona, Italy; 2 Department of Agriculture, Università degli Studi di Sassari, Sassari, Italy; 3 Department of Agricultural, Food and Environmental Sciences, University of Perugia, Perugia, Italy; Aristotle University of Thessaloniki, GREECE

## Abstract

We have investigated the role of selection in the determination of the detected levels of introgression from modern maize hybrid varieties into maize landraces still cultivated *in situ* in Italy. We exploited the availability of a historical collection of landraces undertaken before the introduction and widespread use of modern maize, to analyse genomic changes that have occurred in these maize landraces over 50 years of co-existence with hybrid varieties. We have combined a previously published SSR dataset (n=21) with an AFLP loci dataset (n=168) to provide higher resolution power and to obtain a more detailed picture. We show that selection pressures for adaptation have favoured new alleles introduced by migration from hybrids. This shows the potential for analysis of historical introgression even over this short period of 50 years, for an understanding of the evolution of the genome and for the identification of its functionally important regions. Moreover, this demonstrates that landraces grown *in situ* represent almost unique populations for use for such studies when the focus is on the domesticated plant. This is due to their adaptation, which has arisen from their dynamic evolution under a continuously changing agro-ecological environment, and their capture of new alleles from hybridisation. We have also identified loci for which selection has inhibited introgression from modern germplasm and has enhanced the distinction between landraces and modern maize. These loci indicate that selection acted in the past, during the formation of the flint and dent gene pools. In particular, the locus showing the strongest signals of selection is a *Misfit* transposable element. Finally, molecular characterisation of the same samples with two different molecular markers has allowed us to compare their performances. Although the genetic-diversity and population-structure analyses provide the same global qualitative pattern, which thus provides the same inferences, there are differences related to their natures and characteristics.

## Introduction

Introgressive hybridization has major roles in the evolution of plant populations and in the development of novel diversity and adaptive scenarios [1–6]. Among the different consequences of introgression, such as increased nucleotide diversity, transfer or origin of adaptation, and creation of ecotypes and species, introgression might also facilitate genetic assimilation, with the extinction of populations [7,8].

Novel combinations of genes arising from hybridization can represent new sources of variation on which selection might work. The issue of adaptive introgression due to hybridization as ‘an evolutionary stimulus’ was introduced in the 1950s [1,2,9,10]. There are numerous examples of this in the literature for viruses, bacteria, and plant and animal species, and on the evolution of wild species (see [11] for review), transgene escape [12–14], and crop-weedy-wild introgression [15–21]. These have shown the potential for the identification of functionally important regions of the genome through studies focused on hybridization.

Considering crop plants, the dynamic conservation of landraces can exploit the occurrence of introgression as a novel source of diversity, even if the level and direction of introgression might have an important role in favouring adaptive processes or might result in reduction in genetic diversity (e.g., asymmetric introgression) [16]). At the same time, landraces are an almost unique population for use for such studies when the focus is on the domesticated plant. Indeed, for landraces, the isolation by distance model is often as valid as it is for wild populations [22–23].

In particular the analysis of recent well-defined events like the introduction of modern varieties can be exploited to study gene flow and selection, especially if samples that were collected during different periods are available, such as historical collections. To the best of our knowledge, only two studies in Europe have reported on gene flow analysis over time that have involved landraces and modern varieties: one in barley [23] and the other in maize [24].

The present study is based on the previous study of Bitocchi et al. [24], where changes were compared at the genome level for two collections of maize landraces from the Marche region that were established at two different times; i.e., one recently (from 2000–2005), and the other before the introduction and spread of the cultivation of modern maize hybrid varieties (from the early 1950s). Bitocchi et al. [24] also included flint and dent modern maize hybrid genotypes (FMM, DMM, respectively) in their analyses, as well as landraces from northern Italy. On this basis, they indicated that the recent maize landraces originated and evolved from the gene pool of landraces cultivated in the Marche region (central Italy) before the introduction of the hybrids, and that hybridization events between these landraces and modern varieties has occurred. Thus, from population-structure, diversity, and linkage-disequilibrium analyses, clear and significant levels of introgression into the recent landraces from the modern hybrids was shown.

By genotyping a subsample of these individuals (including only the accessions from 2000; accessions collected in 2001–2005 were not included) using amplified fragment-length polymorphism (AFLP) molecular markers and using the required *simple sequence repeat* (SSR) data from Bitocchi et al. [24], the present study aimed to: (i) investigate the role of selection in the determination of the detected level of introgression in recent landraces after 50 years of co-existence with the cultivation of maize hybrid varieties; (ii) identify loci that show effects of selection that might have been important during the formation of the flint and dent gene pools, or for fitness and adaptation; and (iii) compare the data obtained with these two molecular markers.

## Materials and Methods

### Plant materials

Here, 104 accessions of maize were analysed for a total of 218 genotypes. In particular, five populations characterised the whole sample, as materials shared with those used in the study of Bitocchi et al. [24] (Table 1): two collections of flint maize landraces from the Marche region (Italy) that were collected at two different times, as one in the early 1950s, and thus before the introduction of maize hybrids (old landraces; OLs), comprising 43 accessions (farmers’ fields) with 83 genotypes (individuals), and the other from the year 2000 (recent landraces; RLs; the accessions collected in 2001–2005 in Bitocchi et al. [24] were not included), comprising 20 accessions with 77 genotypes; a set of traditional maize landraces from northern Italy (NI), which were used as parents for the development of the flint hybrid varieties; and sets of both flint (FMM) and dent (DMM) modern maize varieties that each included modern hybrids and inbred lines. Thus, we included the same OL, NI, FMM and DMM populations as Bitocchi et al. [24], with the RLs in the present study as a subsample of those of Bitocchi et al. [24]. The list of accessions used, along with details of the accession codes, numbers of genotypes per accessions, local names for the accessions and pedigrees for the hybrids and inbred lines, and the collection sites of the landraces from the Marche region, are reported in S1 Table. The details of the collections from the Marche region are in Bitocchi et al. [24]. No permits were required for the described collections as the locations are not protected in any way, and endangered or protected species are not involved in the present study.

**Table 1:** Number of accessions and genotypes analysed in this study.

Population	Population code	No. of accessions	No. of genotypes
Old (early 1950s) flint landraces from Marche	OL	43	83
Recent (2000) flint landraces from Marche	RL	20^a^	77^a^
Northern Italy landraces	NI	11	22
Modern flint maize	FMM	8	12
Modern dent maize	DMM	22	24
**Total**		**104**	**218**

^a^The control accession ANGRMC13 (4 genotypes) was included in the RL population.

In the RL population, an accession was included (ANGRMC13) that was not a landrace, but was instead a dent hybrid that had been bought by one of the farmers some years ago and has been cultivated *in situ* to date. The ANGRMC13 RL population was thus used only in the population structure analysis, as a control.

The maize inbred lines used are among the most important elite breeding materials that have been developed by public institutions for use in temperate regions.

### Genotypic data

The genotypes were analysed using 21 SSRs from the study of Bitocchi et al. [24], and using 168 AFLP markers in the present study. The DNA used for the AFLP genotyping was the same as for the study of Bitocchi et al. [24], which was obtained from young leaves using the miniprep-extraction method of Doyle and Doyle [25]. The details for the microsatellite markers, amplification conditions, and genotyping are reported in Bitocchi et al. [24]. The complete SSR dataset is also available from the Dryad Digital Repository [26].

The AFLP protocol was as described by Vos et al. [27], with minor modifications. Briefly, digestion of the total genomic DNA (300 ng) from each accession was carried out using the *Eco*RI (5’-G^AATTC-3’) and *Mse*I (5’-T^TAA-3’) restriction enzymes. The digested products were ligated to the *Eco*RI (5`-CTCGTAGACTGCGTACC-3`; 3`-CTGACGCATGGTTAA-5`) and *Mse*I (5`-GACGATGAGTCCTGAG-3`; 3`-TACTCAGGACTCAT-5`) adapters by incubation at 37°C for 4 h, followed by 20 min at 65°C, and a final temperature of 10°C. The DNA from the restriction–ligation reactions was diluted 10-fold prior to amplification, and pre-selective amplification was performed using the primers *Eco*RI (5`-GACTGCGTACCAATTC-3`) and *Mse*I (5`-GATGAGTCCTGAGTAA-3`), with a single selective nucleotide (*Mse*I+A). These pre-selective reactions were performed in 50 μL total volume, containing 5 μL diluted DNA from the restriction–ligation reactions, 75 ng of each primer, 0.2 mM dNTP, 1.5 mM MgCl_2_, 1× Taq polymerase buffer, and 1 U Taq DNA polymerase (Promega). Amplifications were conducted with a Perkin-Elmer 9700 thermal cycler (PE Applied Biosystems), using touch-down PCR. This involved one cycle of 94°C for 45 s, 65°C for 30 s, and 72°C for 1 min, followed by 12 cycles of 94°C for 30 s, 64.4°C to 56.0°C for 30 s, decreasing at 0.7°C with each cycle, and 72°C for 1 min, and then 20 cycles of 94°C for 30 s; 56°C for 30 s, 72°C for 1 min, and final extension at 72°C for 10 min. The pre-selective amplification products were diluted 1:10 and used as templates with primers, each with three selective nucleotides; the *Eco*RI primers were labelled with the Cy5 dye. S2 Table gives the AFLP primer combinations used and the number of loci scored for each combination. The pre-selective reactions were performed in 20 μL total volume, containing 5 μL diluted DNA from pre-selective reactions, 30 ng of each primer, 0.2 mM dNTP, 1.5 mM MgCl_2_, 1× Taq polymerase buffer, and 1 U Taq DNA polymerase (Promega). The amplification cycle was the same as that described above for the pre-selective reactions. The AFLP fragments were separated on 6% polyacrylamide gels by electrophoresis for 2 h at 50 W constant power, using a Genomix system (Beckman, Fullerton, CA). The scoring for absence and presence of fragments was performed manually. Bands of equal fragment size were assumed to be homologous. To minimise the effects of size homoplasy, only fragments of the medium/ large size classes were scored [28]. Only clearly amplified bands were retained for the analysis (bands with variable intensity across genotypes were not considered). To obtain reliable data, replicated samples were used to investigate polymorphism within the gel, and control genotypes were used to align the different gel runs for each primer combination. The complete AFLP dataset is available as S1 File.

### Genetic diversity and divergence analysis

For the SSR data, the effective number of alleles per locus (n_e_; [29]) and the unbiased expected heterozygosity (He; [30]) were estimated for each population, using the PopGene version 1.32 software [31]. The population genetics package Arlequin version 3.5 [32] was used to estimate: (i) the inbreeding coefficient (F_IS_; [33]) for each population; and (ii) the divergence between the populations, through F_ST_ [33].

To take the dominant different nature of the AFLP markers into account, we first computed allelic frequencies using a Bayesian method, with non-uniform prior distribution of allele frequencies [34] using the AFLP-SURV 1.0 programme [35]; in this analysis, the inbreeding coefficient (F_IS_) computed with the SSRs was integrated as a measure of deviation from the Hardy-Weinberg equilibrium. This method has been shown to produce almost unbiased estimates of allelic frequencies in dominant markers [36]. The second step was to use the estimated allelic frequencies to compute n_e_ [29] and He [30] for each population, and the F_ST_ [33] between populations. Differences between maize landrace populations from the Marche region for the n_e_ and He estimates were tested using the non-parametric Wilcoxon signed-ranks test for two groups; i.e., pairs of estimates for each locus [37].

A Mantel test [38] was performed to test the correlation between the F_ST_ matrices obtained with the SSR and AFLP molecular markers, using the GenAlEx version 6.5 software [39].

### Population structure

The population structure of the whole sample considered was investigated using STRUCTURE version 2.3.4 [40]. For K from one to eight, 20 independent runs were performed, using 30,000 burn-in periods, 30,000 Markov chain Monte Carlo repetitions, and no prior information, and assuming correlated allele frequencies and admixture. For the AFLP, the procedure applied took into account appropriately the genotypic ambiguity inherent to dominant markers [41]. The *ad-hoc* statistic ΔK [42] was used to infer the number of populations (K). The percentage of membership (q) of each genotype in each of the inferred K populations was estimated by one final run for 100,000 burn-in periods and Markov chain Monte Carlo repetitions. The percentage of membership of each accession was computed by averaging the q values of the genotypes belonging to the same accession. A non-parametric correlation analysis (Spearman's correlation coefficient rho [ρ]) was performed to test the relationship between the q values obtained with the SSR and AFLP molecular markers. The Wilcoxon–Kruskal–Wallis non-parametric test was used to test the differences among the accessions for the average percentage of membership (q). The JMP 7 software (SAS Institute, Cary, USA) was used for both of these analyses.

The STRUCTURE data were used to further subdivide the RL accessions into two subgroups: RL_A and RL_B, as the accessions that showed low and high introgression, respectively, from the modern maize. For these subsets, the genetic diversity statistics (n_e_ and He) were used for a comparison with the OL population.

### Selection analysis

Two different methods were used to detect SSR and AFLP loci that were affected by selection during maize evolution. In particular, this analysis was conducted for pairs of populations (e.g., OL-RL, OL-DMM, RL-DMM). The NI and FMM populations were not considered in this analyses because of their small sample size. For the SSR markers, we first applied the method of Beaumont and Nichols [43], as further developed by Beaumont and Balding [44], and as implemented in the FDIST2 software. This approach is based on an island model and simulates the distribution of the F_ST_ conditioned on the heterozygosity, under the null hypothesis of drift and migration only. This has been shown to be relatively robust in different demographic scenarios [44], especially for population pairs [45]. The procedure used for the SSRs was the same as that described by Bitocchi et al. [24]. The AFLP markers were analysed using the same approach, modified for dominant markers; the DFDIST software was used. This programme implements the Bayesian method of Zhivotovsky [34] to estimate allele frequencies. In DFDIST, the loci with a frequency of the most common allele ≥0.98 were excluded; thus, a mean ‘neutral’ F_ST_ value was calculated after trimming 30% of the highest and lowest F_ST_ values. The software simulated the evolution of 100,000 neutral loci under a symmetrical island model with two demes (populations) exchanging migrants. To determine the putative neutral F_ST_, this process was iterated, excluding all of the loci that showed departure from the simulated expected neutral distribution at each following run, until no further locus fell outside the expected distribution. Then, a final run that included all of the AFLP markers was performed to detect loci under selection using the putative neutral F_ST_ estimated. A stringent probability level of significance (P <0.01) was used in each test of neutrality, to avoid type I errors (i.e., the risk of false positives).

Different studies have suggested the use of two or more outlier detection methods to avoid false conclusions [46–48]. Thus, we applied a second approach, as proposed by Foll & Gaggiotti, [49], and implemented in Bayescan version 2.1 [49–50], which directly estimates the probability that each locus is subjected to selection using a Bayesian method. Briefly, the programme defines two alternative models: one that includes the effects of selection, and another that excludes them. The probability of each model was estimated for each locus using a reversible-jump Markov chain Monte Carlo approach [49]. The following default parameters were used: 20 pilot runs of 5,000 iterations, an additional burn-in of 50,000 iterations, followed by 100,000 iterations with a sample size of 5,000 and thinning interval of 10. For the AFLP, a uniform distribution of F_IS_ between 0 and 1 was also used, and the loci with the commonest allele at a frequency ≥0.98 were excluded. The data are expressed for each locus as a Bayes factor (BF), which corresponds to the posterior probability of the selection model with selection over that without it (neutral). The Bayes factors can be converted into a scale of evidence for the effects of selection on the locus [51], as: barely worth mentioning; substantial; strong; very strong; and decisive. For a locus to be an outlier, the ‘very strong’ and ‘decisive’ levels were considered.

The variations in the genetic diversity, He, between the OL and RL populations (ΔH_OL-RL_) and between the two subgroups of the RL population (ΔH_OL-RL_A_ and ΔH_OL-RL_B_) were calculated as ΔH = 1 − (He_OL_/He_RL_), where He_OL_ and He_RL_ are the genetic diversities in the OL and RL populations, respectively [52]. This was carried out separately for neutral loci and for those detected as outliers.

### Sequencing of AFLP locus 26d

Both of the outlier detection methods indicated the AFLP locus ‘26d’ as strongly affected by selection; thus, we sequenced this DNA fragment. Eight different maize accessions were selected, four carried the 26d fragment (OL accessions VA334 and VA331; RL accessions ANGRMC56 and ANGRMC5), and four were characterised by the absence of the 26d fragment (dent hybrid variety TEVERE; the A632 and B73 dent inbred lines; the RL ANGRMC52 accession). One individual for each accession was genotyped, with the protocol performed as described above for the AFLP genotyping. The data (as presence/ absence of the 26d fragment) were consistent with the initial score, and thus the fragments for the four genotypes carrying the 26d band were cut from the polyacrilamide gel using a sterile blade, eluted into a tube with 100 μl double-distilled water, and incubated at room temperature for 24 h. Two microlitres of the elution from each cut fragment was used as the template for PCR amplification, with the same AFLP primer combination that originated the fragment, and under the same conditions, in 100 μL reaction mix. The PCR products were purified using GFX PCR DNA and Gel Band Purification kits (GE Healthcare, UK), according to the manufacturer instructions. The samples were sequenced on both strands using forward and reverse primers with the cycle sequencing reaction with BigDye Terminator Cycle Sequencing Ready Reaction kits (Applied Biosystems, Foster City, CA, USA). The products were resolved on an ABI Prism 3100-Avant automated sequencer (Applied Biosystems, Foster City, CA, USA). Pregap4 and Gap4 of the Staden Software Package (http://staden.sourceforge.net/) were used for the sequence analysis. The Pregap4 modules were used to prepare the sequence data for assembly (quality analysis). Gap4 was used for the final sequence assembly of the Pregap4 output files (normal shotgun assembly). The sequences obtained were aligned using MUSCLE version 3.7 [53] and edited using BIOEDIT version 7.0.9.0 [54]. BLASTn [55] analysis was carried out against the nucleotide collection (nr/nt) NCBI/ GenBank database (http://blast.ncbi.nlm.nih.gov/Blast.cgi, as accessed on 4 June, 2014). A BLASTn analysis was also performed against the sequence database of the B73 Reference Genome assembly (B73 RefGen_v2) (executed at MaizeGDB, http://blast.maizegdb.org/home.php?a=BLAST_UI, as accessed on 31 July, 2014).

The inbred lines ANGRMC56 and A632 that are characterised by the presence and absence of the 26d fragment, respectively, were chosen to carried out reciprocal crosses (A632 × ANGRMC56; ANGRMC56 × A632). The F_1_ genotypes were screened for the presence/ absence of the 26d fragment.

## Results

### Genetic diversity and population divergence

The effective number of alleles per *locus* (n_e_) and the unbiased expected heterozygosity (He) were computed to estimate the levels of genetic diversity for the five populations considered: OL, RL, NI, FMM and DMM. This was carried out using both the SSR and AFLP datasets. The results were very consistent across these two molecular markers (Table 2). In particular, as for the study of Bitocchi et al. [24], we focused our attention on the comparison between the OL and RL maize landrace populations, with the aim being to determine changes that have occurred over the last 50 years, thus from before the introduction of maize hybrids. The n_e_ estimates for RL were higher than for OL for both the SSR (n_e(OL)_ = 3.22 vs. n_e(RL)_ = 3.36; P = 0.006) and AFLP (n_e(OL)_ = 1.37 vs. n_e(RL)_ = 1.43; P <0.0001) datasets. The same was seen for He (SSR: He_(OL)_ = 0.55 vs. He_(RL)_ = 0.63; P = 0.0007; AFLP: He_(OL)_ = 0.23 vs. He_(RL)_ = 0.27; P <0.0001).

**Table 2:** Genetic diversity estimates for the populations analysed using the SSR and AFLP datasets.

Dataset	Population	n_e_	He	F_IS_
SSR	OL	3.22	0.55	0.23
	RL	3.36	0.63	0.37
	RL_A^a^	2.90	0.57	0.33
	RL_B^b^	3.04	0.63	0.36
	NI	3.82	0.65	0.34
	FMM	2.81	0.58	0.28
	DMM	3.30	0.66	0.80
AFLP	OL	1.37	0.23	/
	RL	1.43	0.27	/
	RL_A^a^	1.39	0.24	/
	RL_B^b^	1.44	0.27	/
	NI	1.44	0.27	/
	FMM	1.44	0.28	/
	DMM	1.47	0.29	/

n_e_, Effective number of alleles per locus [29]; He, Unbiased expected heterozygosity [30]; F_IS_, Inbreeding coefficient [33].

Subgroups of the RL population as determined by the population STRUCTURE analysis:

^a^RL_A, accessions showing no or low level of introgression from modern maize;

^b^RL_B, accessions showing high level of introgression from modern maize.

For further population codes, see Table 1.

The highest inbreeding coefficient was that of DMM (F_IS_ = 0.80) (Table 2); this was expected, as almost all of these accessions were inbred lines. Compared to the OL population, the RL population showed a significantly higher F_IS_ (1.61-fold; P = 0.005; Table 2), which can be explained by the fragmentation, which increased the probability of allele fixation within farmers’ subpopulations, and as a consequence, the level of inbreeding; this has occurred over the last 50 to 60 years, due to reductions in the cultivation of traditional landraces and their field sizes, which has led to a reduction in the effective population size of the landraces at the meta-population level.

**Fig 1:**
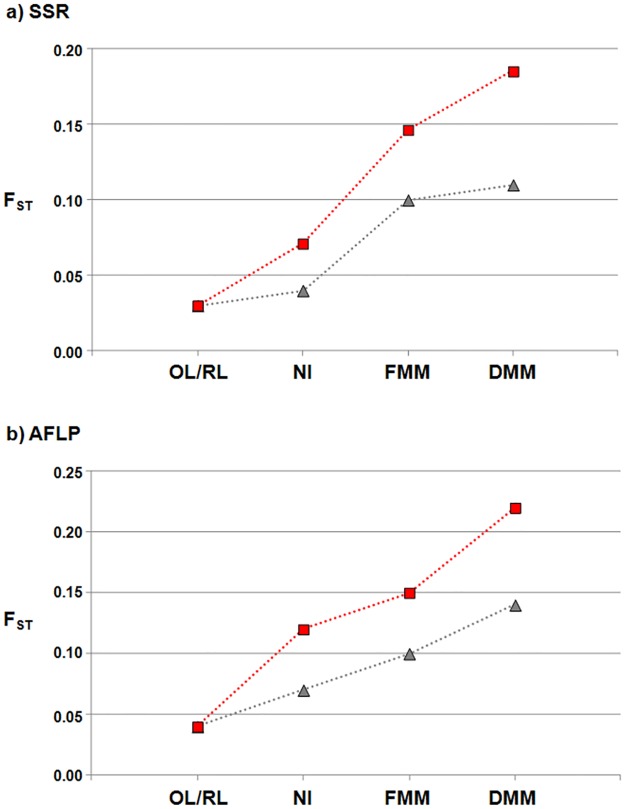
Divergence between populations. Pairwise F_ST_ values between the OL and the RL, NI, FMM and DMM (red squares) populations, and between the RL and the OL, NI, FMM and DMM (grey triangles) populations for the SSR **(a)** and AFLP **(b)** molecular markers.

Estimates of differentiation (F_ST_) between the pairs of populations computed with the SSR and AFLP datasets are reported in S3 Table. The Mantel test showed that the matrices obtained with the two molecular markers were almost identical (r^2^ = 0.87; P = 0.01; S1 Fig.). The lowest differentiation was between the OL and RL populations (F_ST_ = 0.03 and 0.04, for SSR and AFLP, respectively; Fig. 1 and S3 Table). While consistent with the data of Bitocchi et al. [24] that were obtained only with SSRs, also for the AFLP dataset, the genetic differentiation between OL and the other populations increased from NI (F_ST_ = 0.12) to FMM (F_ST_ = 0.15), to a maximum F_ST_ of 0.22 for DMM. This trend was similar considering the differentiation between RL and the other populations, although all of the F_ST_ values were lower compared to those obtained for OL, with the estimates between the OL and the NI, FMM and DMM populations being, on average, 1.6-fold higher that those between the RL and the NI, FMM and DMM populations (Fig. 1 and S3 Table).

### Population structure

The plot of the average *ln likelihood* values over 20 runs for K values ranging from 1 to 8 showed that the *ln likelihood* estimates increased progressively as K increased (S2 Fig.). Thus, we used the *ad-hoc* statistic ΔK [42] to infer the number of populations that characterise our sample. These data were consistent for both SSRs and AFLPs, and suggested that this sample was made up of two main genetic groups, or clusters (S2 Fig.).

**Fig 2:**
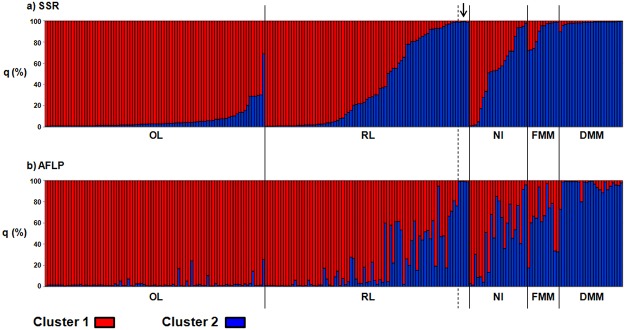
Population structure at individual level. Percentages of membership to cluster 1 (q_1_, red) and cluster 2 (q_2_, blue) for each of the 218 genotypes for the SSR **(a)** and AFLP **(b)** molecular markers. Each genotype is represented by a vertical bar divided into two coloured segments, the lengths of which indicate the proportions of the genome attributed to cluster 1 and cluster 2. The arrow indicates the four genotypes of the control accession ANGRMC13.

The percentages of membership (i.e., q values) for each genotype in each of these two clusters (hereafter referred to as cluster 1 and cluster 2) were computed (Fig. 2). The correlation analysis between the q values between the two different molecular markers indicated that they were highly consistent (Spearman's ρ = 0.84, P <0.0001).

Focusing on the average percentages of membership of each of the five populations in each of these two clusters (q_1_ for cluster 1, q_2_ for cluster 2), the OL population was assigned to cluster 1 (q_1_ = 0.94 and 0.97, for SSRs and AFLPs, respectively); the RL population was mostly assigned to cluster 1 (q_1(SSR)_ = 0.63, q_1(AFLP)_ = 0.79), while the NI population was intermediate between clusters q_1_ and q_2_ (q_1(SSR)_ = 0.45, q_1(AFLP)_ = 0.53) (Table 3). The DMM population was assigned to cluster 2 (q_2_ = 0.98 and 0.95, for SSRs and AFLPs, respectively), as was the FMM population (q_2(SSR)_ = 0.89, q_2(AFLP)_ = 0.62) (Table 3).

**Table 3:** Average membership coefficients to each of the two identified clusters for the populations analysed using the SSR and AFLP datasets.

Dataset	Population	q_1_	q_2_
SSR	OL	0.94	0.06
	RL^a^	0.63	0.37
	NI	0.48	0.52
	FMM	0.11	0.89
	DMM	0.02	0.98
AFLP	OL	0.97	0.03
	RL^a^	0.79	0.21
	NI	0.53	0.47
	FMM	0.38	0.62
	DMM	0.05	0.95

q_1_, Average percentage of membership to Cluster 1; q_2_, Average percentage of membership to Cluster 2.

^a^The control accession ANGRMC13 was excluded by this computation.

For population codes, see Table 1.

**Fig 3:**
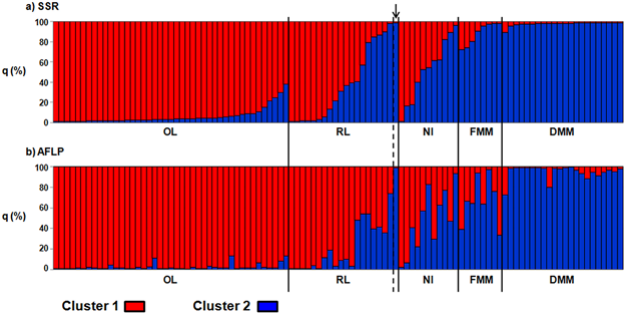
Population structure at accession level. Average percentages of membership to cluster 1 (q_1_, red) and cluster 2 (q_2_, blue) for each of the 104 accessions for the SSR **(a)** and AFLP **(b)** molecular markers. Each accession is represented by a vertical bar divided into two coloured segments, the lengths of which indicate the proportions of the genome attributed to cluster 1 and cluster 2. The arrow indicates the control accession ANGRMC13.

The average percentages of membership at the accession level are reported in Fig. 3; the OL accessions were assigned to cluster 1 with high percentages of membership, which ranged from a minimum q_1(SSR)_ of 0.62 and q_1(AFLP)_ of 0.87, to a maximum q_1(SSR-AFLP)_ of 0.99 (Fig. 3). Moreover, the average q_1_ values of the OL accessions were uniform (Wilcoxon–Kruskal–Wallis non-parametric test; P_SSR_ = 0.08 and P_AFLP_ = 0.09). Similarly, the DMM accessions were assigned to cluster 2 with high values of q_2_ (q_2(SSR)_ from 0.90 to 0.99; q_2(AFLP)_ from 0.73 to 0.99) (Fig. 3). As suggested by Bitocchi et al. [24] from their consideration of only the SSR dataset, the AFLP dataset confirmed that the applied population structure analysis can clearly discriminate between the landraces cultivated before the introduction of maize hybrids (OL) and the modern maize germplasm (DMM). This thus provides a powerful approach for the study of introgression from modern maize varieties into RL landraces, which should be proportional to the q_2_ values. The level of introgression of the RL accessions was highly variable, with the means of their q_1_ values being significantly different (Wilcoxon–Kruskal–Wallis non-parametric test; P <0.0001 for both the SSR and AFLP datasets; control accession ANGRMC13 excluded). To evaluate the different levels of introgression for the RL accessions (no/ low introgression, or admixture/ high introgression), a threshold value of q_1_ was defined for both of the molecular markers, as the lowest average values of q_1_ among the OL accessions (0.62 for SSR, and 0.87 for AFLP). Twelve of the RL accessions (63%) showed no or low introgression from modern maize (RL_A), while the remaining seven RL accessions were admixed or showed high levels of introgression (RL_B). As expected, the control RL accession ANGRMC13, which was not a landrace, but a dent hybrid that had been cultivated *in situ* for some years, was assigned to cluster 2 (q_2_ = 0.99 for both SSR and AFLP; Fig. 3).

The genetic diversity of RL_A was significantly lower than that of RL (comparisons between n_e_ and He estimates of RL and RL_A, P <0.05), but not significantly different from that of the OL population for the SSRs (P >0.30). Considering a significance level of 1%, this was true also for the AFLPs (P >0.01) (Table 2). The genetic diversity estimates of RL_B were similar to those of RL (comparisons between n_e_ and He estimates of RL and RL_B, P >0.05), while they were significantly higher than those of the OL population (P <0.01), with an exception being the comparison between the n_e_ estimates, which did not reach significance (P = 0.06) (Table 2).

### Selection

Tests of neutrality were carried out for three pairs of populations: OL–RL, OL–DMM and RL–DMM. As in Bitocchi et al. [24], this procedure was applied not only to avoid bias related to heterogeneity in demographic parameters among subpopulations [45], but also to determine the loci that were detected as outliers due to selection acting between the flint or dent varieties (i.e., the OL–DMM and RL–DMM comparisons) or to changing environments or favouring new alleles introduced by migration from hybrids over the last 50 years of *in-situ* cultivation (i.e., the OL–RL comparison).

Two different approaches were used to test for selection, for both the SSR and AFLP molecular markers. The Beaumont and Nichols [43] approach was implemented in the FDIST2 software, and this detected only one SSR locus (umc1634) as an outlier in the OL–DMM comparison, at a significance level of 0.01 (Table 4 and S3 Fig.). The second approach was that proposed by Foll and Gaggiotti [49] and implemented in the Bayescan software, which allowed the identification of three loci (bnlg1746, bnlg1094, bnlg1523) as putatively under selection, all of which were detected in the OL–DMM comparison, with bnlg1523 also detected in the OL–RL comparison. However, these loci showed different intensities of evidence of selection in terms of the Jeffrey [51] scale. Considering the OL–DMM comparison, the level of evidence of selection was ‘substantial’ for locus bnlg1746, ‘strong’ for bnlg1094, and ‘decisive’ for bnlg1523; in the OL–RL comparison, bnlg1523 showed little sign of selection (‘barely worth mentioning’ for the Jeffrey [51] scale) (Table 4).

**Table 4:** Summary of the neutrality test results.

Dataset	Ch	Locus	OL-RL		OL-DMM		RL-DMM	
			FDIST2^a^/ DFDIST^b^	Bayescan^c^	FDIST2^a^/ DFDIST^b^	Bayescan^c^	FDIST2^a^/ DFDIST^b^	Bayescan^c^
SSR	2	bnlg1746				2		
	9	umc1634			X			
	7	bnlg1094				3		
	3	bnlg1523		1		5		
AFLP	/	11c	X					
	/	15 d	X					
	/	25 d	X					
	/	26 d			X	4	X	2
	/	8f	X					
	/	33f	X		X			
	/	39f	X					
	/	40f	X					
	/	45f	X					

^a^For SSR dataset

^b^For AFLP dataset

^c^Jeffrey’s interpretation [51], 1, barely worth mentioning (Bayes factor [BF], 1–3); 2, substantial (BF, 3–10); 3, strong (BF, 10–32); 4, very strong (BF, 32–100); 5, decisive (BF, >100);

X, locus detected as outlier by FDIST/ DFDIST (P <0.01);

For population codes, see Table 1.

Ch, Chromosome.

**Fig 4:**
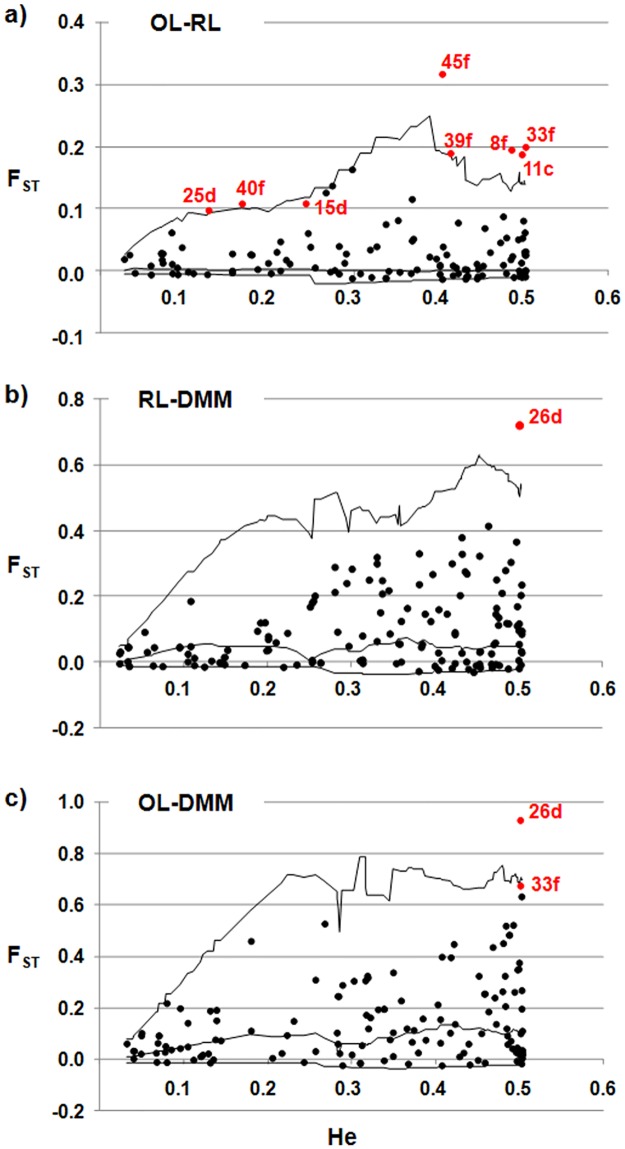
Pairwise comparisons performed with DFDIST using the AFLP markers. Plot of F_ST_ values against heterozygosity estimates for the OL–RL **(a)**, RL–DMM **(b)** and OL–DMM **(c)** populations pairs. Each dot indicates an AFLP locus (black dot, neutral locus; red dot, outlier locus).

A total of nine AFLP loci were detected as outliers by the DFDIST analysis (significance level, 0.01) (Table 4 and Fig. 4). Eight loci (11c, 15d, 25d, 8f, 33f, 39f, 40f, 45f) were detected as under selection in the OL–RL comparison, and two (26d, 33f) in the OL–DMM comparison, with 26d being detected also in the RL–DMM comparison. Bayescan analysis identified locus 26d as an outlier, with very strong evidence of selection in the OL–DMM comparison, and only slight evidence in the RL–DMM comparison. Thus, both of these different methods provided strong evidence of selection for the AFLP locus of 26d; moreover, this locus showed very high differentiation between the landraces and the modern maize (F_ST(OL-DMM)_ = 0.93; F_ST(OL-DMM)_ = 0.72) (Fig. 4).

Table 5 gives the allelic frequencies of the AFLP fragment (band presence) and unbiased expected heterozygosity (He) of the AFLP loci detected as outliers (by one or both of the methods applied) for the OL, RL, NI, FMM, DMM, RL_A and RL_B populations. Considering the eight loci detected in the OL–RL comparison, their allelic frequencies were intermediate between those of the OL and DMM populations, and also between the RL_A and RL_B populations; the frequencies of loci in the RL_A and RL_B populations were closer to the OL and DMM populations, respectively (Table 5). The only exception was locus 45f, which in the RL population had a lower frequency than in the OL, DMM, FMM and RL-A populations (Table 5), while it showed the lowest frequency for the RL_B population, and an allelic frequency in the NI population that was similar to RL. Locus 26d, which was detected as under selection in the comparison between the landraces (OL/ RL) and the modern maize (DMM), showed allelic frequencies that were extreme and opposite for the landraces and dent populations (Table 5). For all of these loci, there was an increase in genetic diversity in RL compared to OL (Table 5). In particular, the variation of the genetic diversity, He, between the OL and RL populations (ΔH_OL-RL_) for the loci detected as under selection in the OL–RL comparison was significantly higher than that computed for the neutral loci (Table 6). This also held true considering the ΔH values between OL and the two subgroups of the RL population (RL_A, RL_B) (Table 6). No significant differences were found in the ΔH values computed for the neutral and outlier loci detected in the comparisons between the landraces (OL/ RL) and the modern maize (DMM; Table 6).

**Table 5:** Frequency of the AFLP fragment (presence of the band) and unbiased expected heterozygosity (He) of the AFLP loci detected as outliers.

Dataset	Locus	Comparison^a^	Population						
			OL	RL	NI	FMM	DMM	RL_A^b^	RL_B^c^
AFLP fragment	11c	OL-RL	0.81	0.49	0.56	0.07	0.11	0.54	0.39
frequency	15d	OL-RL	0.07	0.29	0.43	0.55	0.83	0.22	0.34
	25d	OL-RL	0.02	0.17	0.16	0.13	0.70	0.07	0.38
	8f	OL-RL	0.86	0.54	0.66	0.28	0.29	0.63	0.39
	39f	OL-RL	0.95	0.70	0.86	0.76	0.58	0.89	0.46
	40f	OL-RL	0.03	0.21	0.38	0.21	0.41	0.14	0.36
	45f	OL-RL	0.98	0.70	0.66	0.92	0.92	0.93	0.42
	33f	OL-RL/ OL-DMM	0.76	0.42	0.34	0.28	0.00	0.74	0.04
	26d	OL-DMM/ RL-DMM	0.98	0.87	0.43	0.55	0.05	0.97	0.70
Unbiased expected	11c	OL-RL	0.32	0.51	0.50	0.13	0.20	0.51	0.49
heterozygosity (He)	15d	OL-RL	0.14	0.43	0.50	0.51	0.28	0.35	0.46
	25d	OL-RL	0.03	0.29	0.27	0.23	0.43	0.14	0.48
	8f	OL-RL	0.25	0.51	0.46	0.41	0.42	0.48	0.49
	39f	OL-RL	0.10	0.43	0.24	0.37	0.50	0.21	0.51
	40f	OL-RL	0.06	0.34	0.48	0.34	0.50	0.25	0.47
	45f	OL-RL	0.04	0.43	0.46	0.16	0.15	0.13	0.50
	33f	OL-RL/ OL-DMM	0.37	0.50	0.46	0.41	0.00	0.39	0.07
	26d	OL-DMM/ RL-DMM	0.04	0.24	0.50	0.51	0.10	0.05	0.43

^a^Comparison in which the loci were detected as outliers.

^b^RL_A, accessions showing no or low level of introgression from modern maize;

^c^RL_B, accessions showing high level of introgression from modern maize.

For further population codes, see Table 1.

**Table 6:** Variations in the genetic diversity (He) between the populations (ΔH), computed for the neutral and outlier loci detected in the given comparisons.

	Neutral loci	Outlier loci	P^a^
**ΔH**_OL-RL_	0.12	0.63	0.000
**ΔH**_OL-RL_A_	0.04	0.48	0.001
**ΔH**_OL-RL_B_	0.12	0.64	0.003
**ΔH**_OL-RL_	0.12	0.44	0.096
**ΔH**_OL-RL_A_	0.04	0.08	0.482
**ΔH**_OL-RL_B_	0.12	0.18	1.000

^a^Significance between ΔH values for neutral and outlier loci (Wilcoxon–Kruskal–Wallis non-parametric test).

For population codes, see Table 1.

**Fig 5:**
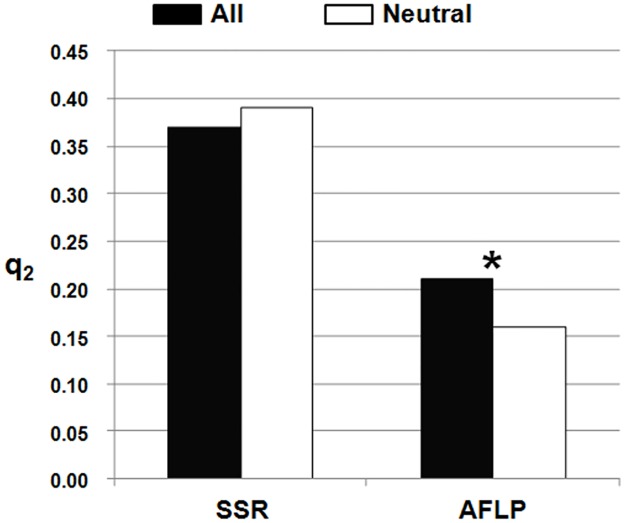
Introgression from modern maize into landraces. Level of introgression from modern maize into the RL population (q_2_ values) computed using only the neutral loci. For the comparison, the q_2_ values were standardised based on the upper q_2_ value (q_2_ of the DMM population) and the lower q_2_ value (q_2_ of the OL population). *, P <0.05.

A neutral dataset was built for both the SSRs and AFLPs by excluding the loci that showed even a minimal effect of selection (all of the loci that also showed minimal signs of selection are reported in Table 4). The level of introgression of this neutral dataset (q_2_ estimate for RL) was then computed. This analysis was carried out to estimate the level of introgression from modern maize into landraces that was not inflated by selection, and thus to determine whether selection had a relevant role in determining the introgression detected. The SSRs and AFLPs gave different results here. In particular, the level of introgression in the RL population computed with the SSRs indicated a similar q_2_ value for the neutral loci (q_2_ = 0.39) and all of the loci (q_2_ = 0.37) (Wilcoxon–Kruskal–Wallis non-parametric test, P = 0.36; Fig. 5). The AFLP results indicated a significantly lower level of introgression with the neutral loci (q_2_ = 0.16) than with all of the loci (q_2_ = 0.21) (Wilcoxon–Kruskal–Wallis non-parametric test, P = 0.04; Fig. 5).

The neutrality test results were also used to identify loci that showed strong signals of selection that might have been important during the formation of the flint and dent gene pools, or for fitness and adaptation. Among the SSR loci, none were detected as under selection by either of the two methods applied (SSRs, AFLPs); however, the locus umc1634 was detected as an outlier with high probability by FDIST2 (significance level, 0.01), and similarly, a strong signal of selection (maximum on the Jeffrey [51] scale) was found also for the bnlg1523 locus by Bayescan. Both of these loci were detected in the OL–DMM comparison. Among the AFLPs, locus 26d showed very strong evidence of selection, as it was detected as an outlier by both methods in comparisons between the landraces (OL/ RL) and the modern maize (DMM). Moreover, this locus showed very high levels of differentiation (F_ST_) between the landraces and the modern maize (F_ST(OL-DMM)_ = 0.93, and F_ST(RL-DMM)_ = 0.72); such high F_ST_ are very unusual in allogamous species like maize, thus, we decided to sequence the 26d AFLP fragment.

The sequence of the 26d locus (total, 170 bp) was obtained by removing the adapter sequences and reconstructing the restriction sites. No polymorphisms were identified between the 26d sequences for the four genotypes used. The sequence has been deposited in the GenBank database (accession number KP406595).

**Fig 6:**
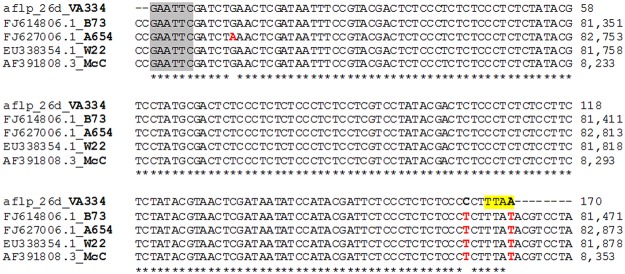
BLASTn analysis. Alignment of the 26d locus sequence and the four best matching sequences from BLASTn searches. Grey, *Eco*RI restriction site; yellow, *Mse*I restriction site.

Through BLASTn [55] searches against the nucleotide collection (nr/nt) database (NCBI/GenBank), we identified four *Zea mays* sequences that showed high similarity with the 26d locus sequence (Fig. 6 and S4 Table). This analysis indicated that the 26d AFLP fragment is part of a DNA transposon, which is a member of the CACTA family, known as *Misfit* [56]. The sequences of the lines W22 [57] and McC [56] were from the genomic region characterised by the *bronze* (*bz*) locus in maize, which is located on the short arm of chromosome 9 (9S). The *Misfit* transposon sequence of the A654 line is from a genomic region that is characterised by the *delta zein* (*dzs10*) gene, which is located on chromosome 9 [58]. In contrast, as showed by Fu and Dooner [56], the B73 line does not carry the corresponding genomic region with the *Misfit* transposon in the *bronze* locus; however, for the 26d fragment, we found correspondence in the B73 line (identity, 99%; e-value, 7e^-81^), with a region located on chromosome 1: the so-called *pcluster* [59]. Several hits for all of the chromosomes characterised by high e-values (from 3.49e^-80^ to 3.47e^-85^) were found by the BLASTn analysis performed against the B73 Reference Genome sequence database. This was expected, considering that the 26d fragment is a partial sequence of a transposable element. As for four sequences found in the NCBI nr/nt database and the B73 Reference Genome, the 26d fragment that was characteristic of individuals from the OL and RL populations was characterised by two mutations (Fig. 6). One mutation was within the restriction site of *Mse*I (‘TTAA’ for 26d, and ‘TTAT’ for all of the other sequences found by BLASTn analysis); this explains the presence of the band for 100% and 96% of the OL and RL individuals, respectively, and its absence in 96% of the DMM varieties and lines. The other polymorphism was a mutation 3 bp upstream of the *Mse*I restriction site (a ‘C’ base, instead of ‘T’).

Screening for the presence/ absence of the 26d fragment in the parental genotypes ANGRMC56 and A632 (characterised by the presence and absence of the 26d fragment, respectively) and in 10 F_1_ genotypes deriving from their reciprocal crosses, indicated that all of the F_1_ genotypes carried the 26d fragment. This excluded the possibility of maternal inheritance for the 26d locus (i.e., chloroplast or mitochondrial genome), which is instead present in the nuclear DNA.

## Discussion

In this study, we have combined the SSR dataset obtained by Bitocchi et al. [24] with AFLP markers, through which we have been able to characterise the role of selection in determining the detected level of introgression into maize landraces from hybrids. This was not evident in the previous study of Bitocchi et al. [24] because of the lower number of loci analysed (i.e., 21 SSRs) compared to the addition here of 168 AFLPs, along with the same 21 SSRs. This was possible because a larger proportion of the genome was covered, which thus increased the probability to include loci linked to regions under selection [60].

However, from the comparison of the genetic diversity and the population structure analyses conducted with the SSR and AFLP datasets for our sample, the data were comparable with the use of this different kind of molecular marker (AFLPs), which confirms the picture highlighted by Bitocchi et al. [24] relating to the evolution of maize landraces from central Italy over the last 50 years, after the introduction and spread of modern hybrids.

### SSRs *versus* AFLPs

One of the aims of this study was to compare the data obtained by the analysis of the same samples with two different molecular markers: SSRs and AFLPs. Even if the genetic diversity and population structure analyses gave the same global qualitative pattern, there remain some differences, which are related to the different natures and characteristics of these different DNA marker systems (i.e., dominant for SSRs, and co-dominant for AFLPs).

The genetic diversity estimates for SSRs were higher than those for AFLPs. This is related to the different mutation rates that are characteristic of these two molecular markers. The mutation rate is higher for SSRs compared to AFLPs [61–63], which results in higher allele variation for SSRs. Moreover, it is important to consider that the He calculated using dominant markers can vary from 0 to 0.5, with the maximum reached when the frequencies of absence and presence of a band are equal (0.5). This consideration clearly indicates that it is not possible to directly compare absolute values of genetic diversity on data from different kinds of markers, particularly when the comparisons are made with dominant *versus* co-dominant markers [64].

The significant correlations between the SSR and AFLP matrices of F_ST_ indicate that both of these marker systems support the same biological inferences. Nevertheless, the F_ST_ estimates computed with AFLPs was slightly higher than those obtained in the SSR analysis. This was also shown by Woodhead et al. [65] in a study that compared SSR and AFLP performances in an analysis of the population genetics structure in *Athyrium distentifolium*. This might be related to the higher number of AFLP loci, which leads to a high probability of including loci linked to regions under selection, thus increasing the differentiation between the populations [60]. Another explanation might be the polymorphic nature of the SSR loci, which are less likely to reach fixation, compared to AFLPs, and for individual loci to have an F_ST_ estimate of 1.

The consistency seen in the present study between the results from the STRUCTURE analysis with the SSR and AFLP data are in agreement with a previous simulation [42], which showed that with the STRUCTURE programme, 100 AFLP loci gave results similar to 10 SSR loci in the detection of the real number of populations. At the individual assignment level, we observed a similar trend, although the AFLP molecular markers were more efficient than the SSR loci in the discrimination of the source of an individual among putative populations; indeed, the individuals were assigned lower percentages of membership (q) to the respective groups with the SSR dataset than the AFLP dataset. This lower discriminating power of SSRs compared to AFLPs was also reported by Campbell et al. [66] in whitefish (*Coregonus clupeaformis*), Garoia et al. [64] in *Solea vulgaris*, and Woodhead et al. [65] in *A*. *distentifolium*. In particular, the lower discriminating power of SSRs compared to AFLPs is due to the lower number of loci that are generally analysed compared to AFLPs, and it is greater when the sample analysed has a low structure [67]. These data indicate that in such analyses that involve individual-based population assignment methods, AFLP molecular markers are particularly useful in systems characterised by weak population structuring, and also when allogamous species are considered.

### Introgression and selection

Due to the combined effects of selection and recombination, it is possible to detect the signature of selection from variant patterns of allelic frequencies, as compared with neutral expectation [68,69]. Therefore, we used different approaches [44,49] to identify loci that showed outlier behaviour for both of the molecular markers, and we used this information in two ways: for the building of a neutral dataset to investigate the genome-wide effects; and to identify loci with strong signals of selection that might mark genomic regions that have been controlling important agronomic traits and/ or that contribute to local adaptation [45,46].

Our data based on SSRs only suggested that selection did not influence the introgression from hybrids into landraces, which was mainly due to a neutral scenario. Moreover, the two SSR loci detected with high signals of selection between the OL and DMM populations suggest a selection taking place in the past during the evolution of the flint and dent types, and in any case, before the introduction of the hybrids. However, the analysis conducted with AFLPs allowed a more detailed scenario; indeed, the estimation of introgression into the recent maize landraces was significantly lower for neutral compared to all of the AFLP loci, which indicated a role of selection in determining introgression from hybrids into maize landraces. Moreover, for the AFLPs, most of the selection signatures were detected between OL and RL, with putative AFLP loci under selection showing in RL, frequencies that were intermediate between OL and DMM. All of these data indicate that in part, positive selection influenced the level of introgression observed in the RL populations by favouring alleles donated by DMM. The loci detected as outliers in the comparisons between the OL and RL indicate that selection pressures for adaptation have favoured new alleles introduced by migration from hybrids over the last 50 years. This shows the critical role of migration in the evolution of landrace populations grown on farms.

There have been few studies based on investigations of historical adaptive introgression in cultivated species. Recently, Hufford et al. [21] documented patterns of genome-wide introgression between maize and populations of its wild relative *Z*. *mays* ssp. *mexicana* that were grown in sympatry in Mexico. They provided evidence of the presence of adaptive alleles from *Z*. *mays* ssp. *mexicana* into the maize genome that were incorporated during its expansion to the highlands of central Mexico. In rice, the introgression that has been shown for different groups of cultivated rice has involved genes that control important agronomic traits [18,19,70].

Along with these studies, our findings show the potential of analysing historical introgression, and even within as short a time period as 50 years, to provide further understanding of their evolution and to identify functionally important regions of the genome. Moreover, our study provides evidence of the great opportunities offered by landraces to reach these goals. Indeed, landraces are characterised by adaptation that derives from a historical dynamic evolution (under continuous pressures of different evolutionary forces) that is not only related to genetic diversity created by new mutations, but also to the capture of new alleles from hybridization. In particular, as suggested by Barton [71], such introgression can lead to adaptation at considerably higher rates compared to those for non-hybridizing populations. This is clearly shown by our data, where signatures of adaptive introgression were found for this very short time of co-evolution of landraces with modern maize (50 years). The possibility of using landrace collections taken at different times (closely related populations) further enhances the efficiency of genome scans for divergent selection due to the lower effects of (i) mutations, that are less likely to obscure a potential selective footprint; and (ii) drift on the genetic diversity parameters used to infer loci putatively under selection (see [72]).

A further outcome of our study is related to the identification of loci that show effects of selection in the comparisons between the landraces and the modern maize. In particular, we found two loci: one, locus 33f, was detected as an outlier in the OL–DMM comparison; and the other, locus 26d, was identified in the OL–DMM and RL–DMM comparisons. The allelic frequencies of these two loci were extreme and opposite for the landraces (especially for locus 26d) and dent populations; this suggests that for these loci, selection has inhibited introgression from modern germplasm (negatively selected in the RL landraces). Moreover, as we have seen for the two SSR loci that showed the strongest signals of selection (umc1634, bnlg1523), and which are located in genomic regions that carry important genes in the control of the starch characteristics in the kernels, they enhance the distinction of the landraces from the modern maize. This suggests that the selected loci were important loci during the formation of the flint and dent gene pools, and that they have probably experienced purifying selection by the farmers, either consciously or unconsciously. The high values of F_ST_ between the landraces and the modern maize for these loci (F_ST_ = 0.72 and 0.93, for 33f and 26d, respectively) are very unusual for neutral loci in an allogamous species like maize, also because of the low relevance of hitchhiking, due to the rapid decay of linkage disequilibrium. Thus, our data suggest that our two AFLP loci are tagging a genomic segment that is very close (a few kb) to the phenotypically causative molecular variant that is associated to the differential fitness in the two populations. However, the very high divergence observed for these loci might have an alternative explanation. Indeed, a locus with maternal inheritance (i.e., the chloroplast or mitochondrial genomes) might show the same departure of allelic frequencies from that expected for nuclear neutral loci, by assuming that: (i) the polymorphism reflects an old diagnostic polymorphism that was alternatively fixed after the formation of the flint and dent gene pools; and (ii) introgression from hybrids has occurred mostly by pollen flow.

We focused our attention on the 26d locus, which showed the strongest evidence of selection pressures. For this locus, the molecular characterisation of the parents characterised one for the presence and the other for the absence of the 26d fragment, and of F_1_ genotypes derived from reciprocal crosses between them. Along with the sequencing of the 26d fragment, this allowed us to establish that this locus is present in the nuclear genome, and not in the chloroplast or mitochondrial genomes, and thus maternal inheritance can be excluded. BLASTn analysis against the B73 Reference Genome indicated high similarity of the 26d fragment sequence in all of the maize chromosomes, so it was not possible to pinpoint the exact position of our fragment in the maize genome. After all, it is well-known that maize is characterised by a highly polymorphic genome structure [73,74], with interspersion of genes and retrotransposons that varies from line to line. Nevertheless, taking this aspect carefully into account, we found high similarities with sequences of three maize inbred lines located on chromosome 9, in particular those for the W22 and McC lines were located in the genomic region characterised by the *bronze* (*bz*) locus in maize, on the short arm of chromosome 9 (9S). This genomic region in maize has several genes that control visible kernel traits, including *Colourless kernel 1* (*C1*), *Shrunken 1* (*Sh1*), *Bronze 1* (*Br1*) and *Waxy* (*Wx*), and it has been shown that the size of the *bz1* region can vary by more than three-fold among maize lines [75]. Thus, our data suggest that a deeper investigation of this region in our landraces is needed, to identify a possible functional role in the evolution of maize landraces for the *Misfit* transposable element located on the short arm of chromosome 9, particularly in the distinction between the flint and dent maize types. There is evidence in the literature that indicates that transposable element activity has had a key part in adaptive evolution (for review, see [76]). For example, in maize, Studer et al. [77] showed recently that a transposable element inserted in a regulatory region of the *teosinte branched 1* (*tb1*) gene, which is one of the major domestication genes in maize, acts as an enhancer of gene expression, and partially explains the increased apical dominance in maize, compared to its progenitor, teosinte.

## Conclusions

Genome scans using large numbers of randomly selected markers to reveal loci that deviate from neutral expectations, such that they might represent genomic regions that contribute to important traits [45,46], have been widely used in different studies (for reviews, see [78,79]) including those focused on crop species (e.g. [80–82]). Several of these have been based on AFLP data in both animal and plant species, to identify loci that show the potential of the approach, especially in non-model organisms (for review, see [83]). The identification of outlier AFLP loci in the present study is further evidence of the usefulness and reliability of these methods.

The present study is one of the few that have been aimed at investigating historical adaptive introgression in cultivated species [18,19,21,70]. One of the major key factors of the present study is not only the use in the analysis of landraces maintained *in situ*, and thus under agro-ecological conditions that have been continuously changing, but also, in contrast to the studies mentioned above in maize and rice, we had the possibility to study their evolution over the last 50 years, due the availability of collections carried out at different times. Interestingly, we found that adaptation followed by hybridization with modern maize varieties was very fast, with landraces capturing and increasing the frequency of favourable alleles over very short times.

The implications of such studies are numerous, starting from providing a deep understanding of evolutionary processes, to identifying genes/ genomic regions that control phenotypic traits, which is a crucial goal for breeders.

## Supporting Information

S1 Fig Correlation between F_ST_ matrices. Mantel test between the F_ST_ matrices obtained with the SSR and AFLP molecular markers.
(TIF)

S2 Fig Estimation of the number of population (K) that characterizes the sample. Average *ln likelihood* values over 20 runs for increasing K values from 1 to 8 (left), and for ΔK for increasing K values from 2 to 7 (right), for the SSR **(a)** and AFLP **(b)** molecular markers.
(TIF)

S3 Fig Pairwise comparisons performed with FDIST2 using the SSR markers. Relationship between F_ST_ and the heterozygosity estimates for the OL–RL **(a)**, RL–DMM **(b)**, and OL–DMM **(c)** population pairs. Each dot indicates an SSR locus (black dot, neutral locus; red dot, outlier locus).
(TIF)

S1 File AFLP dataset. (XLSX)

S1 Table Details of the accessions used for the analysis. (DOC)

S2 Table AFLP primer combinations used in this study, with number of loci scored for each combination. (DOC)

S3 Table F_ST_ estimates between pairs of populations for the SSR and AFLP markers. (DOC)

S4 Table Best matches of 26d locus sequence against the nr/nt GenBank database using BLASTn searches (accessed 4 June, 2014). (DOC)
