# Supplementary figures and images for: European Flint Landraces Grown In Situ Reveal Adaptive Introgression from Modern Maize
Source: PLoS One. 2015 Apr 8;10(4):e0121381. doi: 10.1371/journal.pone.0121381 (PMC4390310; doi:10.1371/journal.pone.0121381)

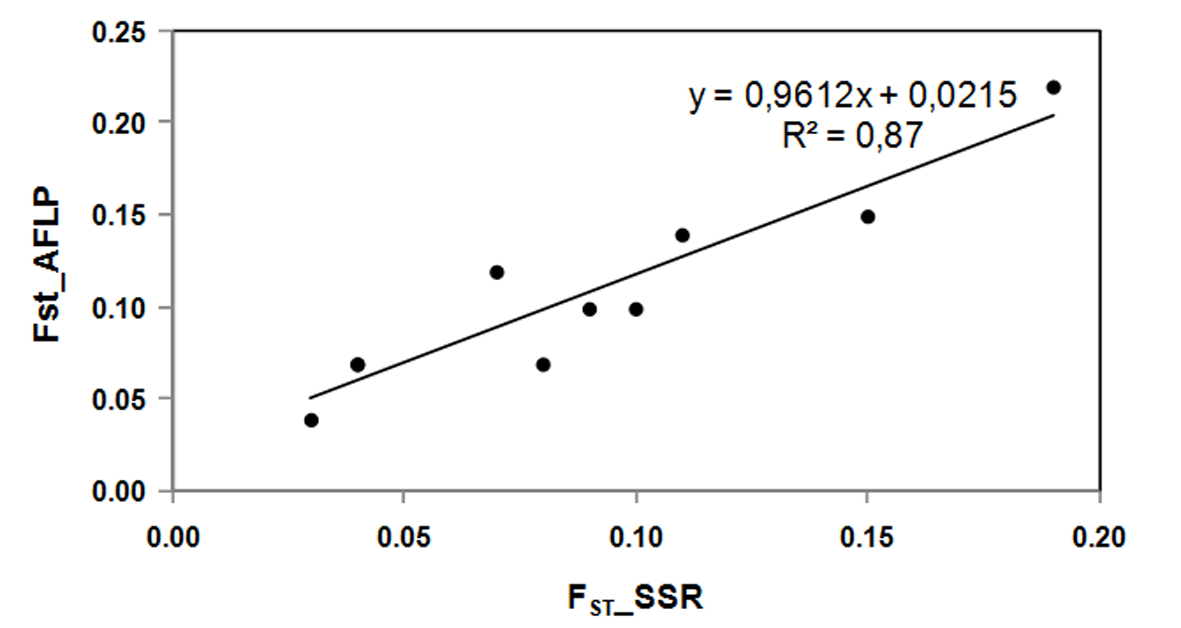

Supplement: S1 Fig — Mantel test between the FST matrices obtained with the SSR and AFLP molecular markers. (TIF) [file pone.0121381.s001.tif]

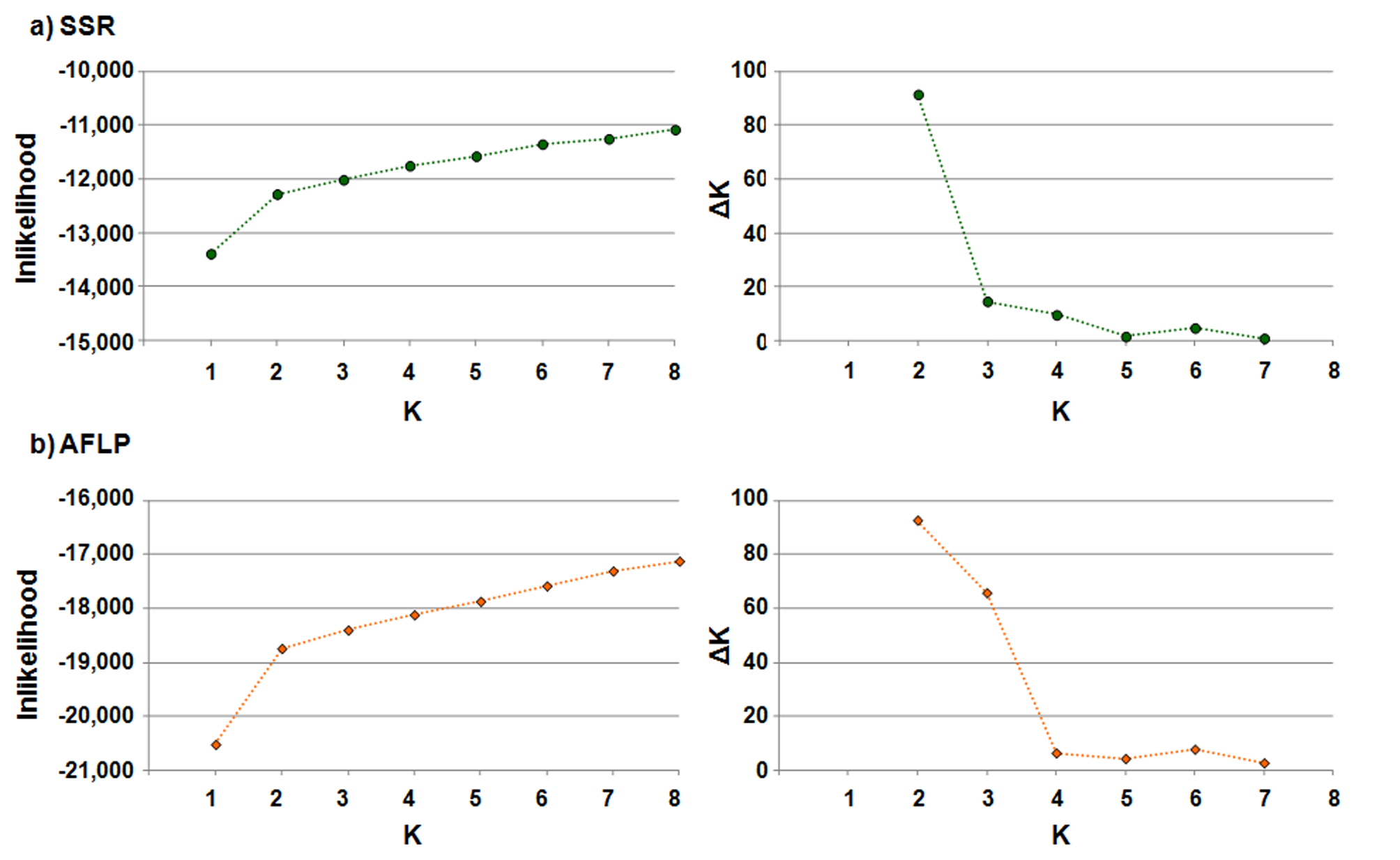

Supplement: S2 Fig — Average ln likelihood values over 20 runs for increasing K values from 1 to 8 (left), and for ΔK for increasing K values from 2 to 7 (right), for the SSR (a) and AFLP (b) molecular markers. (TIF) [file pone.0121381.s002.tif]

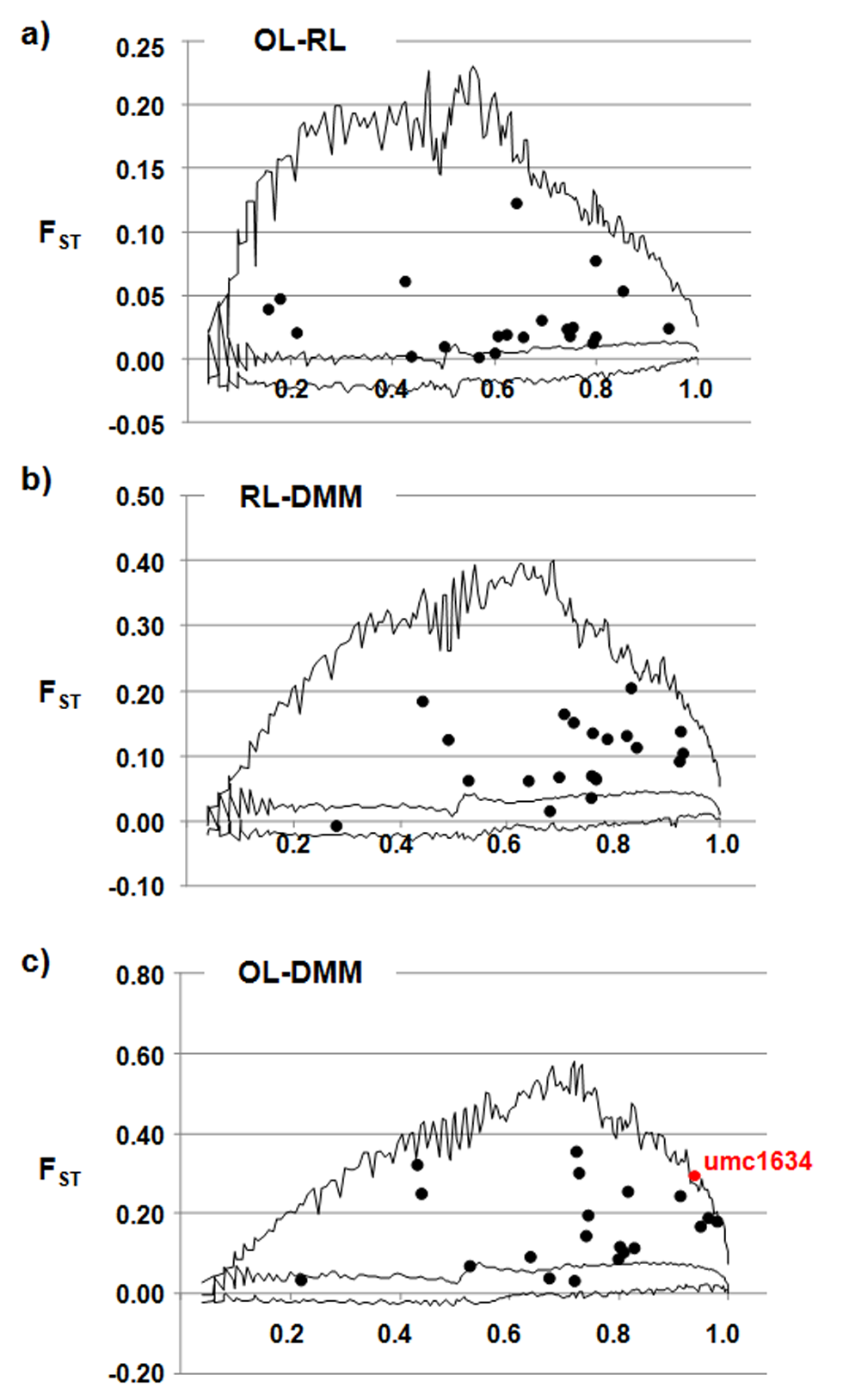

Supplement: S3 Fig — Relationship between FST and the heterozygosity estimates for the OL–RL (a), RL–DMM (b), and OL–DMM (c) population pairs. Each dot indicates an SSR locus (black dot, neutral locus; red dot, outlier locus). (TIF) [file pone.0121381.s003.tif]
