# Supplementary material for: European Flint Landraces Grown In Situ Reveal Adaptive Introgression from Modern Maize
Source: PLoS One. 2015 Apr 8;10(4):e0121381. doi: 10.1371/journal.pone.0121381 (PMC4390310; doi:10.1371/journal.pone.0121381)
Supplement: S1 Table — (DOC) [file pone.0121381.s005.doc]

**Supporting Information Tables**

***Table S1. Details of the accessions used for the analysis.***

| **Accession**  **Code** | **Genotypes**  **per accession** | **Population**  **code** | **Local Name/Pedigree** | **Province or**  **Region/Origin** | **Collection site** |
| --- | --- | --- | --- | --- | --- |
| VA325 | 2 | OL | Cannellino | Ascoli Piceno | Montegiorgio-Piane M. Verde |
| VA326 | 2 | OL | Culaccione | Ascoli Piceno | Montegiorgio-Piane M. Verde |
| VA327 | 2 | OL | Otto file locale | Macerata | S. Ginesio |
| VA328 | 2 | OL | 14 file locali | Macerata | Recanati |
| VA329 | 1 | OL | Quarantino | Macerata | Cingoli |
| VA330 | 2 | OL | Otto file locale | Macerata | Recanati |
| VA331 | 2 | OL | 12 file locale | Macerata | S. Ginesio |
| VA332 | 2 | OL | Otto file maceratese | Macerata | Macerata |
| VA333 | 2 | OL | Nostrano incrociato | Pesaro | Montecerignone-Maisano |
| VA334 | 2 | OL | Locale | Pesaro | Serrungarina |
| VA335 | 2 | OL | Locale | Pesaro | Fano |
| VA336 | 2 | OL | Locale | Pesaro | Urbino |
| VA337 | 2 | OL | Incrocio | Pesaro | Pietrarrubbia Badia |
| VA338 | 2 | OL | Locale | Pesaro | Monteporzio |
| VA339 | 2 | OL | Locale | Pesaro | Pennabilli-Soanne |
| VA340 | 2 | OL | Quarantino | Pesaro | Pergola |
| VA341 | 2 | OL | Locale | Pesaro | Urbino |
| VA342 | 2 | OL | Locale | Pesaro | Novafeltria |
| VA343 | 2 | OL | Locale | Pesaro | Urbino |
| VA344 | 2 | OL | Nostrano incrociato | Pesaro | Macerata Feltria |
| VA306 | 2 | OL | Nostrale | Ancona | Jesi |
| VA307 | 2 | OL | Culaccione | Ascoli Piceno | Offida |
| VA304 | 2 | OL | Quarantino 8 file | Ancona | Putido di Fabriano |
| VA308 | 2 | OL | Granturchella | Ascoli Piceno | Offida-Ciafone |
| VA309 | 1 | OL | Fusello | Ascoli Piceno | Torre San Patrizio |
| VA310 | 2 | OL | Spadone | Ascoli Piceno | Fermo |
| VA346 | 2 | OL | Ideale | Pesaro | Pergola |
| VA311 | 2 | OL | Culaccione | Ascoli Piceno | Torre San Patrizio |
| VA312 | 2 | OL | Agostinello | Ascoli Piceno | Ascoli-Cimigliano |
| VA313 | 2 | OL | Nano | Ascoli Piceno | Acquasanta Falciano |
| VA314 | 2 | OL | Cannellino | Ascoli Piceno | Grottammare |
| VA315 | 2 | OL | Nostrano senza nome | Ascoli Piceno | M. Prandone |
| VA316 | 2 | OL | Spadone | Ascoli Piceno | S. Benedetto del Tronto |
| VA317 | 2 | OL | Culaccione | Ascoli Piceno | Grottammare |
| VA318 | 2 | OL | Nostrano senza nome | Ascoli Piceno | Amandola |
| VA319 | 2 | OL | Nostrano senza nome | Ascoli Piceno | M. Fortino Colle Alto |
| VA320 | 2 | OL | Nostrano senza nome | Ascoli Piceno | Amandola-Marnacchia |
| VA321 | 2 | OL | Nostrano senza nome | Ascoli Piceno | Amandola-Ciaraglia |
| VA322 | 2 | OL | Nostrano senza nome | Ascoli Piceno | S. Vittoria S. Salvatore |
| VA323 | 2 | OL | Nostrano senza nome | Ascoli Piceno | Montegiorgio-S. Susanna |
| VA324 | 2 | OL | Nostrano senza nome | Ascoli Piceno | Servigliano-Parapina |
| VA305 | 2 | OL | Cinquantino | Ancona | Putido di Fabriano |
| VA345 | 2 | OL | Quarantino | Pesaro | Pergola |
| ANGRMC1 | 4 | RL | 2 spighe | Macerata | Vallinfante (Parco dei Sibillini) |
| ANGRMC3 | 3 | RL | Quarantino | Macerata | Villarella (Parco dei Sibillini) |
| ANGRMC5 | 4 | RL | Quarantino | Macerata | Villarella (Parco dei Sibillini) |
| ANGRMC6 | 4 | RL | **/** | Macerata | Villarella (Parco dei Sibillini) |
| ANGRMC7 | 4 | RL | **/** | Pesaro | Frontone |
| ANGRMC8 | 4 | RL | quarantino | Pesaro | Monterolo-Frontone |
| ANGRMC9 | 4 | RL | quarantino | Macerata | Villarella (Parco dei Sibillini) |
| ANGRMC13 | 4 | RL | estero | Macerata | Villarella (Parco dei Sibillini) |
| ANGRMC14 | 4 | RL | **/** | Ascoli Piceno | Monteprandone |
| ANGRMC17 | 4 | RL | quarantino | Ancona | Monterosso (stazione) |
| ANGRMC18 | 4 | RL | nostrale | Ancona | Monterosso (stazione) |
| ANGRMC19 | 4 | RL | mais primitivo | Ancona | Monterosso (stazione) |
| ANGRMC50 | 3 | RL | **/** | Ascoli Piceno | Amandola |
| ANGRMC51 | 3 | RL | **/** | Ascoli Piceno | Cerqueto |
| ANGRMC52 | 3 | RL | **/** | **/** | **/** |
| ANGRMC53 | 4 | RL | **/** | Ascoli Piceno | Montefortino |
| ANGRMC54 | 4 | RL | **/** | Ascoli Piceno | Torre S. lucia Aquasanta |
| ANGRMC55 | 4 | RL | 4dGj polenta | Ascoli Piceno | Pomaro- Aquasanta Terme |
| ANGRMC56 | 4 | RL | 7bGj polenta | Ascoli Piceno | Piedicava-Aquasanta Terme |
| ANGRMC57 | 5 | RL | 4cGj da polenta | Ascoli Piceno | Aquasanta Terme |
| CRAB1 | 2 | NI | Ottofile bianco dell'Albese | Piemonte | **/** |
| CRAB2 | 2 | NI | Ottofile rosso dell'Albese | Piemonte | **/** |
| CRAB3 | 2 | NI | Ottofile giallo di La Morra | Piemonte | **/** |
| CRAB4 | 2 | NI | Pignoletto giallo del Torinese | Piemonte | **/** |
| CRAB5 | 2 | NI | Pignoletto rosso del Canavese | Piemonte | **/** |
| CRAB6 | 2 | NI | Pignoletto rosso dell’Albese | Piemonte | **/** |
| CRAB7 | 2 | NI | Ostenga del Canavese | Piemonte | **/** |
| VA87 | 2 | NI | Nostrano dell'isola | Milano | **/** |
| VA159 | 2 | NI | Marano | Venezia | **/** |
| VA191W | 2 | NI | Bianco perla | Udine | **/** |
| VA558 | 2 | NI | Rostrato | **/** | **/** |
| PR36Y03 | 1 | FMM | PR36Y03 – Dent 2 x Flint Marano 2 | PIONEER | / |
| Zimapan | 2 | FMM | ZIMAPAN – Dent x Flint Bianco perla | VERNEUIL | / |
| Maranello | 1 | FMM | MARANELLO – Dent x Nostrano dell’Isola | VERNEUIL | / |
| Banguy | 2 | FMM | BANGUY – Dent F. x Flint Cornet F. | SIS | / |
| Belgrano | 2 | FMM | BELGRANO – Dent F. x Marano 1 ITA | EMILSEME | / |
| FM802 | 2 | FMM | FM802 – Dent 1 ITA x Flint Marano | FONDAZIONE | / |
| F7 | 1 | FMM | OP Lacaune | NCB | / |
| F2 | 1 | FMM | OP Lacaune | NCB | / |
| Tevere | 2 | DMM | TEVERE – Dent x Dent | DEKALB | / |
| Balka | 2 | DMM | BALKA – Dent x Dent | PIONEER | / |
| A632 | 1 | DMM | [(Mt42*B14)B14(3)] | CCB-B14 | / |
| A619 | 1 | DMM | [(A171*Oh43)Oh43] | CCB-Oh43 | / |
| B14 | 1 | DMM | Cuzco*B14(8) | CCB-B14 | / |
| NC258 | 1 | DMM | TZ(2)*[(NC248*246)*C103] | CCB-C103 | / |
| NC250 | 1 | DMM | [(Nigeria Composite Arb*B37)B37) | CCB-B37 | / |
| N28 | 1 | DMM | Stiff Stalk Synthetic 1 | CCB-SSS | / |
| B73 | 1 | DMM | Iowa Stiff Stalk Synthetic C5 | CCB-B73 | / |
| HP301 | 1 | DMM | Supergold | Pop | / |
| B37 | 1 | DMM | Iowa Stiff Stalk Synthetic | CCB-B37 | / |
| KY21 | 1 | DMM | Boone County White | South | / |
| K55 | 1 | DMM | Pride of Saline | CCB | / |
| F44 | 1 | DMM | Smith(Old Florida variety) | South | / |
| ND246 | 1 | DMM | W755*W771 | NCB | / |
| T8 | 1 | DMM | Jarvis Golden Prolific | South | / |
| WF9 | 1 | DMM | Reid yellow dent (Indiana station strain) | CCB | / |
| OH43 | 1 | DMM | Oh40B*W8 | CCB-Oh43 | / |
| C103 | 1 | DMM | Lancaster Surecrop (from Noah Hershey) | CCB-C103 | / |
| B84 | 1 | DMM | BS13(S2)C0 | CCB-SSS | / |
| GT112 | 1 | DMM | Multiple cross | South | / |
| MO17 | 1 | DMM | C.I.187-2*C103 | CCB-C103 | / |

OL, old landraces; RL, recent landraces; NI, northern Italy landraces; FMM, modern flint maize; DMM, modern dent maize
