# Supplementary material for: European Flint Landraces Grown In Situ Reveal Adaptive Introgression from Modern Maize
Source: PLoS One. 2015 Apr 8;10(4):e0121381. doi: 10.1371/journal.pone.0121381 (PMC4390310; doi:10.1371/journal.pone.0121381)
Supplement: S2 Table — (DOC) [file pone.0121381.s006.doc]

**Supporting Information Tables**

Table S2. AFLP primer combinations used in this study, with number of loci scored for each combination.

| **Number** | **Primer** | | **Code** | **No. of** |
| --- | --- | --- | --- | --- |
|  | ***Eco*RI^a^** | ***Mse*I^a^** |  | **polymorphic loci** |
| 1 | +CTG | +ATA | A | 10 |
| 2 | +TCG | +ATT | B | 27 |
| 3 | +ACG | +ATT | C | 43 |
| 4 | +GAT | +AGG | D | 31 |
| 5 | +AAT | +AAC | F | 57 |
| **Total** | **/** | **/** | **/** | **168** |

^a^EcoRI primer: 5`-GACTGCGTACCAATTC-3`;

^b^MseI primer: 5`-GATGAGTCCTGAGTAA-3`.
