# Supplementary material for: European Flint Landraces Grown In Situ Reveal Adaptive Introgression from Modern Maize
Source: PLoS One. 2015 Apr 8;10(4):e0121381. doi: 10.1371/journal.pone.0121381 (PMC4390310; doi:10.1371/journal.pone.0121381)
Supplement: S3 Table — (DOC) [file pone.0121381.s007.doc]

**Supporting Information Tables**

**Table S3. F_ST_ estimates between pairs of populations for the SSR and AFLP markers.**

| **Dataset** | **Populations** | **OL** | **RL** | **NI** | **FMM** | **DMM** |
| --- | --- | --- | --- | --- | --- | --- |
| **SSR** | **OL** | 0 |  |  |  |  |
|  | **RL** | 0.03 | 0 |  |  |  |
|  | **NI** | 0.07 | 0.04 | 0 |  |  |
|  | **FMM** | 0.15 | 0.10 | 0.08 | 0 |  |
|  | **DMM** | 0.19 | 0.11 | 0.09 | 0.04 | 0 |
| **AFLP** | **OL** | 0 |  |  |  |  |
|  | **RL** | 0.04 | 0 |  |  |  |
|  | **NI** | 0.12 | 0.07 | 0 |  |  |
|  | **FMM** | 0.15 | 0.1 | 0.07 | 0 |  |
|  | **DMM** | 0.22 | 0.14 | 0.1 | 0.07 | 0 |

For population codes, see Table 1.
