# Supplementary material for: European Flint Landraces Grown In Situ Reveal Adaptive Introgression from Modern Maize
Source: PLoS One. 2015 Apr 8;10(4):e0121381. doi: 10.1371/journal.pone.0121381 (PMC4390310; doi:10.1371/journal.pone.0121381)
Supplement: S4 Table — (DOC) [file pone.0121381.s008.doc]

**Supporting Information Tables**

Table S4. Best matches of 26d locus sequence against the nr/nt GenBank database using BLASTn searches (accessed 4 June, 2014).

| **Description** | **GenBank Accession** | **E-value** | **Identity** | **Ch.** | **Reference** |
| --- | --- | --- | --- | --- | --- |
| *Zea mays* cultivar B73 p cluster | FJ614806.1 | 7e-81 | 99% | 1 | [59] |
| *Zea mays* cultivar W22 *bz* gene locus | EU338354.1 | 7e-81 | 99% | 9 | [57] |
| *Zea mays* cultivar McC *bz* locus region | AF391808.3 | 7e-81 | 99% | 9 | [56] |
| *Zea mays* cultivar A654 transposon *Misfit* putative TNP2-like protein and putative TNP1-like protein genes, complete cds; and disrupted dzs10 gene | FJ627006.1 | 8e-80 | 99% | 9 | [58] |

Ch., chromosome
